# New Anti-Inflammatory and Anti-Proliferative Constituents from Fermented Red Mold Rice *Monascus purpureus* NTU 568

**DOI:** 10.3390/molecules15117815

**Published:** 2010-11-03

**Authors:** Ya-Wen Hsu, Li-Chuan Hsu, Chao-Lin Chang, Yu-Han Liang, Yao-Haur Kuo, Tzu-Ming Pan

**Affiliations:** 1 Department of Biochemical Science and Technology, National Taiwan University, Taipei, Taiwan 10617, Taiwan; 2 Division of Herbal Drugs and Natural Products, National Research Institute of Chinese Medicine, Taipei, Taiwan 11221, Taiwan; 3 Graduate Institute of Integrated Medicine, China Medical University, Taichung, Taiwan 40402, Taiwan

**Keywords:** red mold rice (RMR), *Monascus purpureus* NTU 568, azaphilonoid derivatives, anti-inflammatory, anti-proliferative

## Abstract

Six azaphilonoid derivatives, including two new blue fluorescent monapurfluores A (**1**) and B (**2**), two known pyridine-containing molecules, monascopyridines C (**3**) and D (**4**), and two known monasfluores A (**5**) and B (**6**), were isolated and characterized from red mold rice fermented by *Monascus purpureus* NTU 568. Structural elucidation of new isolates was based on nuclear magnetic resonance (^1^H- NMR, ^13^C-NMR, COSY, HMQC, and HMBC) and other spectroscopic analyses. Bioactivity evaluation indicated that **1**-**6** possessed anti-inflammatory activities with dose-dependent relationships for lipopolysaccharide (LPS)-induced nitric oxide production. Furthermore, **1**-**4** also showed moderate antiproliferative effects against human laryngeal carcinoma (HEp-2) (IC_50_ = 14.81~20.06 μg/mL) and human colon adenocarcinoma (WiDr) (IC_50_ = 12.89~21.14 μg/mL).

## 1. Introduction

A growing body of evidence suggests a direct link between inflammation and cancer. It has been found that various steps in tumorigenesis, such as cellular transformation, promotion, proliferation, and metastasis, can be influenced by chronic inflammation [1]. Nitric oxide (NO), a metabolic intermediate induced by activated inflammatory cells, could directly oxidize DNA, resulting in cancer development [2]. Thus, it is well-accepted that anti-inflammatory agents have significant potential pharmaceutical applications in the prevention and treatment of cancer [3]. 

**Figure 1:**
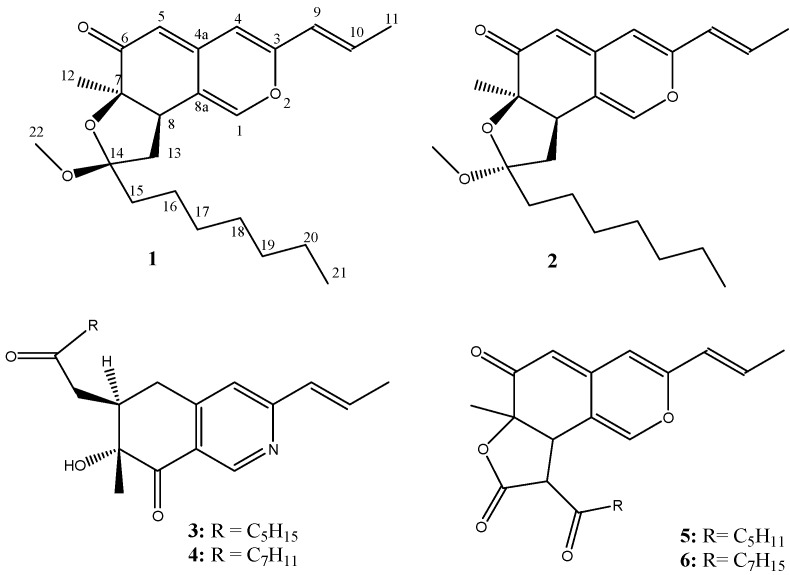
The structures of compounds **1**-**6** (**1**: monapurfluore A:, **2**:, monapurfluore B **3**: monascopyridine C, **4**: monascopyridine D, **5**: monasfluore A, **6**: monasfluore B).

Red mold rice (RMR), a fermented product of *Monascus* species, has been used as a food additive to enhance color and flavor and as a remedy for digestive and vascular diseases in Chinese traditional medicine [4,5]. RMR is also regarded as a health food in Asia and in the United States for its ability to reduce total cholesterol and lipoprotein levels in the liver, an effect caused by one of its components, monacolin K, which is a competitive inhibitor of 3-hydroxy-3-methylglutaryl coenzyme A (HMG-CoA) reductase [6]. The extracts of RMR have been reported to have several *in vitro* pharmacological effects, including antioxidant, anti-inflammatory, and antitumor activities [7,8,9]. Pharmacognosy research has corroborated that *Monascus* species contain several bioactive secondary metabolites, such as monacolins with hypolipidemic activities, γ-aminobutyric acid (GABA) with an antihypertensive effect, dimerumic acid, which reduces the damage caused by oxidative-stress in cells, and azaphilonoid pigments with anti-inflammatory and antitumor activity [10,11,12,13,14]. In our previous studies, *Monascus purpureus* NTU 568 fermented RMR was examined for the regulation of obesity-related factors [15], the mitigation of oral carcinogenesis in 7,12-dimethyl-1,2-benz[a]anthracene (DMBA)-induced oral tumors [16], and the amelioration of memory impairment [17] *in vivo*. Recently, three new yellow pigments, monaphilones A, B and C, were isolated from RMR by our laboratory [18]. Consequently, the aim of this study was the investigation of bioactive components from *M. purpureus* NTU 568 fermented RMR. The isolated compounds **1**-**6** (Figure 1) were assayed for their anti-inflammatory and anti-proliferative activities.

## 2. Results and Discussion

### 2.1. Structure determination

**Figure 2:**
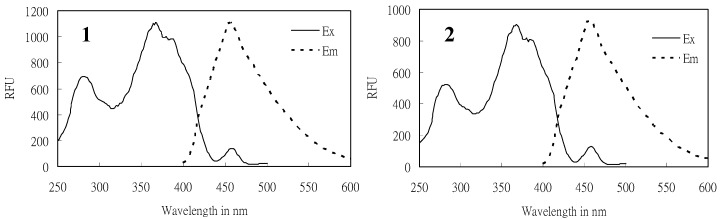
The excitation (Ex) and emission (Em) spectra of **(1)** monapurfluore A and **(2)** monapurfluore B, measured by a fluorescence spectrophotometer. (λ_ex_ = 368 nm, λ_em_ = 456 nm).

Compound **1** was obtained as a slightly yellow oil, and it demonstrated strong blue fluorescence under UV-light irradiation (λ = 365 nm). Its fluorescence spectrum presented maximum excitation and emission at 368 and 456 nm, respectively, as shown in Figure 2, indicating the presence of an extended conjugated system. The HREI-MS of **1** showed a molecular ion at *m/z* 372.2292 [M]^+^, suggesting a molecular formula of C_23_H_32_O_4_, which contains eight required degrees of unsaturation. 

**Figure 3:**
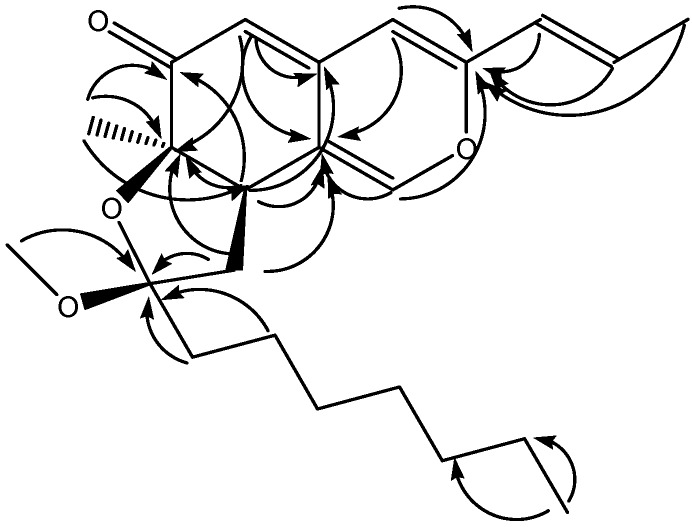
Key HMBC correlations of monapurfluore A.

The IR absorbance bands (1,706, 1,623 cm^-1^) indicated the existence of carbonyl groups and olefinic groups in **1**, and the ^1^H-NMR spectrum of **1** exhibited five olefinic (δ_H_ 5.23, 6.11, 6.15, 6.42 and 7.37), one methoxyl (δ_H_ 3.09), and three methyl (δ_H_ 0.88, 1.19 and 1.85) protons. The ^13^C-NMR spectrum of **1** (Table 1) showed eight olefinic carbons (δ_C_ 107.5, 108.6, 118.6, 124.4, 133.8, 142.8, 144.8 and 155.2) and a conjugated carbonyl carbon (δ_C_ 195.7). Three singlet olefinic protons (5.23, 6.15 and 7.37) in the ^1^H-NMR spectrum, along with HMBC long-range correlations (H-1/C-3 and C-8a, H-4/C-3 and C-8a, H-5/C-4a, C-7 and C-8a and H-8/C-4a, C-6, C-7 and C-4a) (Figure 3), suggested a typical isochroman-6-one azaphilone skeleton [19]. Compound **1** had a *trans*-propenyl moiety, as shown by the coupling constant between H-9 and H-10 (*J* = 15.6 Hz) and the COSY cross peaks of H-9/H-10 and H-10/H-11. Based on the HMBC correlations between H-9/C-3 and between CH_3_-12/C-6, C-7 and C-8, the *trans*-propenyl and CH_3_-12 groups were assigned at C-3 and C-7, respectively. The above corroboration, together with the number of degrees of unsaturation, allowed us to assign **1** to have a pendant ring. The nature of the ring was determined by the COSY correlation between H-8/H-13, and the HMBC correlation between H-13/C-7 and C-14, indicating that **1** possessed a five-membered furan ring fused to C-7 and C-8 of the azaphilonoid structure. Moreover, cross peaks from H-15 to H-21 were observed in the COSY spectrum of **1**, proving the presence of a heptyl side chain. In the HMBC spectrum of **1**, correlations between H-15/C-14 and OCH_3_-22/C-14 were found; thus, both the alkyl side chain and OCH_3_-22 were located at C-14 of the five-membered furan ring.

**Table 1:** ^1^H-NMR (400 MHz) and ^13^C-NMR (100 MHz) spectroscopic data for monapurfluore A (**1**) and monapurfluore B (**2**) (δ in *d*_6_-acetone). ^a,b^

No.	1			2	
	δ_H_	δ_C_		δ_H_	δ_C_
1	7.37 (s)	144.8		7.47 (s)	145.5
3		155.2			155.5
4	6.15 (s)	107.5		6.16 (s)	107.4
4a		142.8			143.4
5	5.23 (s)	108.6		5.22 (s)	107.8
6		195.7			196.0
7		83.4			84.0
8	3.07 (t, *J* = 13.2 Hz)	45.6		3.31 (dd, *J* = 12.8, 7.2 Hz)	44.4
8a		118.6			117.9
9	6.11 (d, *J* = 15.6 Hz)	124.4		6.11 (d, *J* = 16.0 Hz)	124.2
10	6.42 (dq, *J* = 15.6, 7.2 Hz)	133.8		6.45 (dq, *J* = 16.0, 6.8 Hz)	133.5
11	1.85 (d, *J* = 7.2 Hz)	18.2		1.86 (d, *J* = 6.8 Hz)	18.3
12	1.19 (s)	24.6		1.27 (s)	24.7
13	2.07 (t, *J* = 13.2 Hz)	42.2		1.87 (t, *J* = 12.8 Hz)	43.7
	2.42 (t, *J* = 13.2 Hz)			2.08 (dd, *J* = 12.8, 7.2 Hz)	
14		108.6			108.1
15	1.55 (m)	35.8		1.34 (m)	36.0
	1.77 (m)			1.82 (m)	
16	1.29 (m)	25.0		1.27 (m)	25.0
17	1.29 (m)	30.1		1.27 (m)	30.1
18	1.29 (m)	30.4		1.27 (m)	30.4
19	1.29 (m)	32.5		1.27 (m)	32.4
20	1.29 (m)	23.2		1.27 (m)	23.2
21	0.88 (t, 7.2)	14.3		0.87 (t, 7.2)	14.3
22	3.09 (s)	48.4		3.19 (s)	48.2

*^a^* Assignments were confirmed by ^1^H-^1^H COSY, HMQC, HMBC. *^b^* m: multiplet signal.

The relative configuration of the tetrahydrofuran moiety was established based on a 1D NOE (Nuclear Overhauser Effect, in CDCl_3_) experiment. After the irradiation of H-8, the protons of CH_3_-12 were enhanced, whereas no enhancement was observed for OCH_3_-22, suggesting that the relative configurations between H-8/OCH_3_-22 and between H-8/CH_3_-12 were *trans*- and *cis*-, respectively. Based on this evidence, monapurfluore A (**1**) was determined to be 8-heptyl-9,9a-dihydro-8β-methoxy-6a-methyl-3-[(*E*)-prop-1-enyl]-6a*H*-furo[2,3-*h*]isochromen-6(8*H*)-one, a new natural azaphilone derivative. 

The HREI-MS of **2** displayed a molecular ion at *m/z* 372.2308 [M]^+^, indicating that this compound has the same molecular formula as **1** (C_2__3_H_32_O_4_). The fluorescence, IR, UV, ^1^H-, and^ 13^C-NMR spectra showed that **2** and **1** were azaphilone stereoisomers. The chemical shifts of C-8, C-13 and C-14 in **2** were shifted upfield by ∆δ_c_ 1.2, 1.5 and 0.5 ppm, respectively, compared with **1**. After the irradiation of H-8 in the 1D NOE experiment, enhanced signals for CH_3_-12 and OCH_3_-22 were seen, suggesting that these protons were of the *α*-configuration. Thus, the structure of **2** (monapurfluore B) was established as 8-heptyl-9,9a-dihydro-8α-methoxy-6a-methyl-3-[(*E*)-prop-1-enyl]-6a*H*-furo[2,3-*h*] iso-chromen-6(8*H*)-one. 

Compounds **3**-**6** were identified as the known compounds monascopyridine C, monascopyridine D, monasfluore A and monasfluore B, respectively, by comparison with authentic samples and literature data [20,21].

### 2.2. Inhibitory effects on the proliferation of human cancer lines

Compounds **1**-**6** were evaluated for anti-proliferative activity using the HEp-2 (human laryngeal carcinoma) and WiDr (human colon adenocarcinoma) cell lines, respectively. The evaluations were initially tested at 100 μg/mL and further measured at 50, 25, 12.5 and 6.25 μg/mL to obtain data on the 50% cell growth inhibition (IC_50_). Our results indicated that compounds **1**-**4** showed potential inhibition on HEp-2 and WiDr cell lines with IC_50_ values ranging from 12.89 to 21.14 μg/mL (Table 2); whereas the other known fluorescent compounds, monasfluore A (**5**) and monasfluore B (**6**), did not show any anti-proliferative effects on the tested cell lines. However, monascopyridines was reported to have moderate cytotoxic and antimitotic activities for the immortalized human kidney epithelial (IHKE) cells [22]. Thus, this type of azaphilone derivatives might possess side effects which should be of concern for the further investigation. 

**Table 2:** Anti-proliferation Effects of Compounds **1**-**6** for HEp-2 and WiDr Cell Lines.

Compound	IC_50_ *^a^* of HEp-2	IC_50_ of WiDr
	(μg/mL)	
monapurfluore A	18.82 ± 0.37	20.61 ± 1.77
monapurfluore B	15.45 ± 0.98	13.72 ± 0.45
monascopyridine C	20.06 ± 0.53	21.14 ± 2.00
monascopyridine D	14.81 ± 3.16	15.07 ± 2.51
monasfluore A	-*^b^*	-
monasfluore B	-	-
mitomycin C*^c^*	0.07 ± 0.00	0.13 ± 0.00

*^a^* IC_50_: inhibitory concentration 50%. *^b^* IC_50_ > 100 μg/mL. *^c^* Positive control.

### 2.3. Inhibitory effect on NO production

**Figure 4:**
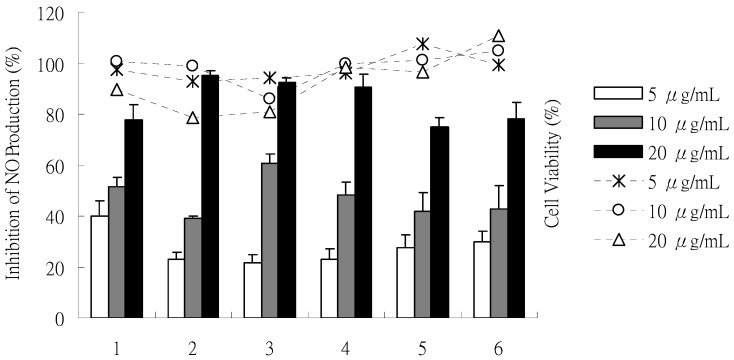
Effects of isolates (**1**-**6**) on NO synthesis in LPS stimulated RAW 264.7 cells (%) and cell viability (%) at concentrations of 5, 10, 20 μg/mL. The bars and the lines indicate inhibition of NO production and cell viability, respectively. (**1**: monapurfluore A, **2**: monapurfluore B, **3**: monascopyridine C, **4**: monascopyridine D, **5**: monasfluore A, **6**: monasfluore B). The results are expressed as the mean ± SD (n = 3) and significantly different from LPS without test compounds (*p* < 0.05).

The effects of compounds **1**-**6** on NO production in an LPS-stimulated RAW 264.7 macrophage are shown in Figure 4. NO accumulation in the culture medium was observed after 24 h for RAW 264.7 cells, stimulated by 1 μg/mL LPS. The MTT assay showed high cell viability (>80%) in the absence or presence of LPS in the culture medium at various concentrations. Compounds **1**-**6** exhibited significant and dose-dependent inhibition of the LPS-stimulated NO production with inhibitory potencies from 20% to 95% at 5, 10 and 20 μg/mL. The IC_50_ values of **1** to **6** were 9.6, 7.8, 9.4, 9.0, 12.8 and 12.4 μg/mL, respectively, compared with positive control, quercetin (IC_50_ = 4.0 μg/mL).

## 3. Experimental

### 3.1. General

Infrared (IR) spectra were taken using a Mattson Genesis II spectrophotometer (Thermo Nicolet, Madison, WI, USA). Optical rotations were determined on a JASCO P-1020 polarimeter. Electrospray ionization mass spectrometry (ESI-MS) data were acquired by a LCQ mass spectrometer (Finnigan MAT LCQ, San Jose, CA, USA). Electronic ionization mass spectrometry (EI-MS) and high resolution electronic ionization mass spectrometry (HREI-MS) were obtained from a FOCUS GC with a DSQ™ II single quadrupole mass spectrometer (Thermo Fisher Scientific Inc. Waltham, MA, USA) and a Finnigan/Thermo Quest MAT-95XL mass spectrometer (Finnigan MAT LCQ, San Jose, CA, USA), respectively. NMR spectra were run on a Bruker NMR spectrometer (Unity Plus 400 MHz) (Brucker BioSpin, Rheinstetten, Germany) and a Varian NMR spectrometer (Unity Plus 600 MHz, Varian Inc., Palo Alto, CA, USA) using acetone-*d*_6_ as the solvent. Sephadex LH-20 (GE Healthcare, Uppsala, Sweden) and silica gel 60 (70-230 mesh and 230-400 mesh, Merck, Darmstadt, Germany) were used as chromatographic materials. Silica Gel 60 F254 plates (Merck) were used for thin layer chromatography (TLC). The TLC spots were detected under UV-lamps (254 and 365 nm) and also by using an anisaldehyde-sulphuric acid solution, applied as a spray reagent, followed by heating. The high performance liquid chromatography (HPLC) was performed using a Shimadzu LC-6AD apparatus with a SPD-6AV UV detector that was equipped with a preparative Cosmosil AR-II column (250 × 20 mm i.d., Nacalai Tesque, Inc., Kyoto, Japan).

### 3.2. Reagents

HPLC grade methanol and analytical grade solvents (acetone, ethyl acetate, *n*-hexane and methanol) were purchased from ECHO Chemical Co. (Miaoli, Taiwan). Anisaldehyde acid and sulphuric acid were purchased from Merck. Fetal bovine serum (FBS), Dulbecco’s minimum essential medium (DMEM), minimum essential medium (MEM), phosphate buffered saline (PBS) solution, and trypan blue were purchased from Biological Industries (Kibbutz Beit Haemek, Israel). Other chemicals, including 3-(4,5-dimethylthiazol-2-yl)-2,5-diphenyltetrazolium bromide (MTT), lipopolysaccharide (LPS), and dimethyl sulfoxide (DMSO), were obtained from Sigma (St. Louis, MO, USA).

### 3.3. Preparation of red mold rice

Long-grain rice (*Oryza sativa*) was fermented by *M. purpureus* NTU 568 as described in our previous report [11]. After a ten-day cultivation, the RMR was further dried and crushed to yield the material for extraction.

### 3.4. Extraction and isolation

The RMR powder (5 kg) was extracted with methanol (25 L) at 50 ºC for 24 h. The solution was then repeatedly percolated through filter paper, and the filtrates were combined and further concentrated under reduced pressure. The dried, red-colored residue was subjected to silica gel column chromatography, eluting with a mixture of *n*-hexane/ethyl acetate (10:0, 9:1, 8:2, 7:3, 6:4, 1:1, 4:6, 0:10), resulting in the collection of eight fractions (Fr. 1-8). After irradiation with UV-light at 365 nm, Fr. 5 showed strong blue fluorescence. This fraction was then further separated by silica gel column chromatography, eluted with *n*-hexane/ethyl acetate (7:3 and 6:4), to give four subfractions (Fr. 5-1 to 5-4). Fr. 5-2 was further chromatographed on a Sephadex (LH-20) gel column to remove other impurities and then purified again using preparative HPLC (Cosmosil 5C_18_ packing column, 250 × 20 mm i.d, MeOH/H_2_O = 85:15, 7 mL/min) to obtain compounds **3** (41.1 mg, at 11.1 min) and **4** (29.6 mg, at 17.4 min). Fr. 5-3 was chromatographed on a Sephadex (LH-20) gel column and a silica gel column, employing a gradient of chloroform/ethyl acetate (96:4, 90:10, 8:2, and 7:3). Finally, compounds **1** (11 mg, at 31.2 min) and **2** (7 mg at 33.5 min) were purified by preparative HPLC (Cosmosil 5C_18_ packing column, 250 × 20 mm i.d, MeOH/H_2_O = 85:15, 7 mL/min) from Fr. 5-3-3-3. Compounds **5** (8 mg at 20.1 min) and **6** (12 mg, at 41.8 min) were obtained from Fr. 5-3-3-4 by preparative HPLC (MeOH/H_2_O, 80:20). 

### 3.5. Spectral data

*Monapurfluore A* (**1**). Slightly yellowish oil; [α]^25^_D_: +18.84 (*c* 0.69, acetone); UV (MeOH) λ_max_: (log ε) 368 (4.1), 284 (3.9); IR: *v*_ma__x_ (KBr) 2,954, 2,926, 2,855, 1,706, 1,623, 1,572, 1,548, 1,453, 1,370, 1,299, 1,255, 1,176, 1,097, 9,18 cm^-1^; EIMS *m/z* 372 [M]^+^; HREI-MS *m/z* 372.2292 [M]^+^ (calc’d for C_23_H_32_O_4_, 372.2301); ^1^H- and ^13^C-NMR data, see Table 1.

*Monapurfluore B* (**2**). Slightly yellowish oil; [α]^25^_D_: -71.43 (*c* 0.42, acetone); UV (MeOH) λ_max_: (log ε) 367 (4.2), 284 (3.9); IR: *v*_ma__x_ (KBr) 2,918, 2,851, 1,714, 1,627, 1,572, 1,548, 1,441, 1,374, 1,310, 1,239, 1,176, 1,093, 914 cm^-1^; EI-MS *m/z* 372 [M]^+^; HREI-MS *m/z* 372.2308 [M]^+^ (calc’d for C_23_H_32_O_4_, 372.2301); ^1^H- and ^13^C-NMR data, see Table 1.

### 3.6. Cell lines and culture conditions

HEp-2 (human laryngeal carcinoma), WiDr (human colon adenocarcinoma), and RAW 264.7 (murine macrophage) were obtained from Food Industry Research and Development Institute (Hsinchu, Taiwan). All cell lines were maintained in MEM containing 5% foetal bovine serum and were kept in a 37 ºC incubator with 5% CO_2_.

### 3.7. Cancer cell growth inhibitory assay

HEp-2 and WiDr were seeded in MEM (180 μL) in 96-well plates (3 × 10^3^ per well). After 4 h, test agents (20 μL), dissolved in PBS solution, were added to reach final concentrations of 6.25, 12.5, 25, 50 and 100 μg/mL . Twenty μL of MTT solution (2 mg/mL) was added to each well and incubated for 4 h in a 37 ºC incubator with 5% CO_2_. After three days of incubation, the cellular conversion of a tetrazolium salt into a formazan product was achieved. The supernatant was removed and DMSO (200 μL) was added to dissolve the formazan, which was finally detected by spectrophotometry at a wavelength of 570 nm, and the relative estimate of cell proliferation was calculated.

### 3.8. Assay of nitrite production

RAW 264.7 cells (5 × 10^4^ per well) were seeded and maintained with DMEM (90 μL) in 96-well plates. After incubating for 12 h, wells were treated with LPS (1 μg/mL) and test agents (10 μg/mL) dissolved in DMEM. The nitrite concentrations of the supernatants were determined using a Griess reagent kit (Promega, Madison, WI, USA) after 24 h. The cell proliferation was evaluated by a cell growth inhibitory assay.

### 3.9. Data analysis

The data on cancer cell growth inhibition and nitrite production were presented as mean ± standard deviation for three independently performed experiments (n = 3). Significant difference was analyzed by Student‘s *t*-test. Differences were considered significant at *p* < 0.05.

## 4. Conclusions

In this study, two new and four known azaphilone derivatives were isolated from *M. purureus* NTU 568 fermented red mold rice. The structures of new azaphilone compounds **1 **and **2 **were elucidated by spectral methods. Bioassays revealed that the isolates **1**-**4** showed moderate anti-proliferation effects against human cancer cell lines HEp-2 and WiDr and anti-inflammatory activity by the inhibition of LPS-induced NO production. The current results, together with the exhibition of anti-NO activity by the crude *M. purpureus* NTU 568 fermented RMR extracts [16], suggest that the azaphilone derivatives in the RMR could play a crucial role in these anti-inflammatory activities.

## References

[1] Aggarwal B.B., Shishodia S., Sandur S.K., Pandey M.K., Sethi G. (2006). Inflammation and cancer: How hot is the link?. Biochem. Pharmacol..

[2] Balkwill F., Mantovani A. (2001). Inflammation and cancer: back to Virchow?. Lancet.

[3] Macarthur M., Hold G.L., El-Omar E.M. (2004). Inflammation and Cancer-II. Role of chronic inflammation and cytokine gene polymorphisms in the pathogenesis of gastrointestinal malignancy. Am. J. Physiol.: Gastrointest. Liver Physiol..

[4] Journoud M., Jones P.J.H. (2004). Red yeast rice: a new hypolipidemic drug. Life Sci..

[5] Ma J.Y., Li Y.G., Ye Q., Li J., Hua Y.J., Ju D.J., Zhang D.C., Cooper R., Chang M.  (2000). Constituents of red yeast rice, a traditional Chinese food and medicine. J. Agr. Food Chem..

[6] Endo A. (1979). Monacolin K, a new hypocholesterolemic agent produced by a *Monascus* species. J. Antibiot..

[7] Lee C.L., Wang J.J., Pan T.M. (2008). Red mold rice extract represses amyloid beta peptide-induced neurotoxicity via potent synergism of anti-inflammatory and antioxidative effect. Appl. Microbiol. Biotechnol..

[8] Lin W.Y., Hsu W.Y., Hish C.H., Pan T.M. (2007). Proteome changes in caco-2 cells treated with *Monascus*-fermented red Mold rice extract. J. Agr. Food Chem..

[9] Ho B.Y., Pan T.M. (2009). The *Monascus* metabolite monacolin K reduces tumor progression and metastasis of Lewis lung carcinoma cells. J. Agr. Food Chem..

[10] Heber D., Yip I., Ashley J.M., Elashoff D.A., Elashoff R.M., Go V.L.W. (1999). Cholesterol-lowering effects of a proprietary Chinese red-yeast-rice dietary supplement. Am. J. Clin. Nutr..

[11] Su Y.C., Wang J.J., Lin T.T., Pan T.M. (2003). Production of the secondary metabolites gamma-aminobutyric acid and monacolin K by *Monascus*. J. Ind. Microbiol. Biotechnol..

[12] Aniya Y., Ohtani I.I, Higa T., Miyagi C., Gibo H., Shimabukuro M., Nakanishi H., Taira J. (2000). Dimerumic acid as an antioxidant of the mold, *Monascus anka*. Free Radic. Biol. Med..

[13] Akihisa T., Tokuda H., Ukiya M., Kiyota A., Yasukawa K., Sakamoto N., Kimura Y., Suzuki T., Takayasu J., Nishino H. (2005). Anti-tumor-initiating effects of monascin, an azaphilonoid pigment from the extract of *Monascus pilosus* fermented rice (red-mold rice). Chem. Biodiv..

[14] Su N.W., Lin Y.L., Lee M.H., Ho C.Y. (2005). Ankaflavin from *Monascus*-fermented red rice exhibits selective cytotoxic effect and induces cell death on Hep G2 cells. J. Agr. Food Chem..

[15] Chen W.P., Ho B.Y., Lee C.L., Lee C.H., Pan T.M. (2008). Red mold rice prevents the development of obesity, dyslipidemia and hyperinsulinemia induced by high-fat diet. Int. J. Obes..

[16] Tsai R.L., Ho B.Y., Pan T.M. Red mold rice mitigates oral carcinogenesis in 7,12-dimethyl-1,2-benz[a]anthracene-induced oral carcinogenesis in Hamster. Evidence-advanced Compl. Alt. Med..

[17] Lee C.L., Kuo T.F., Wang J.J., Pan T.M. (2007). Red mold rice ameliorates impairment of memory and learning ability in intracerebroventricular amyloid beta-infused rat by repressing amyloid beta accumulation. J. Neurosci. Res..

[18] Hsu Y.W., Hsu L.C., Liang Y.H., Kuo Y.H., Pan T.M. (2010). Monaphilones A-C, three new antiproliferative azaphilone derivatives from *Monascus purpureus* NTU 568. J. Agr. Food Chem..

[19] Quang D.N., Stadler M., Fournier J., Tomita A., Hashimoto T., Cohaerins C.F. (2006). Four azaphilones from the xylariaceous fungus *Annulohypoxylon cohaerens*. Tetrahedron.

[20] Knecht A., Cramer B., Humpf H.U. (2006). New *Monascus* metabolites: Structure elucidation and toxicological properties studied with immortalized human kidney epithelial cells. Mol. Nutr. Food Res..

[21] Huang Z.B., Xu Y., Li L.S., Li Y.P. (2008). Two new *Monascus* metabolites with strong blue fluorescence isolated from red yeast rice. J. Agr. Food Chem..

[22] Knecht A., Humpf H.U. (2006). Cytotoxic and antimitotic effects of *N*-containing *Monascus* metabolites studied using immortalized human kidney epithelial cells. Mol. Nutr. Food Res..

